# Osimertinib in the treatment of resected EGFR-mutated non-small cell lung cancer: a cost-effectiveness analysis in the United States

**DOI:** 10.3389/fphar.2024.1300183

**Published:** 2024-03-28

**Authors:** Gengwei Huo, Ying Song, Wenjie Liu, Xuchen Cao, Peng Chen

**Affiliations:** ^1^ Department of Thoracic Oncology, Tianjin Medical University Cancer Institute and Hospital, National Clinical Research Center for Cancer, Key Laboratory of Cancer Prevention and Therapy of Tianjin, Tianjin’s Clinical Research Center for Cancer, Tianjin, China; ^2^ Department of Pharmacy, Jining No. 1 People’s Hospital, Jining, Shandong, China; ^3^ The First Department of Breast Cancer, Tianjin Medical University Cancer Institute and Hospital, National Clinical Research Center for Cancer, Key Laboratory of Cancer Prevention and Therapy of Tianjin, Tianjin’s Clinical Research Center for Cancer, Tianjin, China

**Keywords:** Osimertinib, non-small cell lung cancer, ADAURA, cost-effectiveness analysis, Markov model

## Abstract

**Background:** In the double-blind phase III ADAURA randomized clinical trial, adjuvant osimertinib showed a substantial overall survival benefit in patients with stage IB to IIIA, EGFR-mutated, completely resected non-small cell lung cancer (NSCLC). We conduct a cost-effectiveness analysis comparing the use of adjuvant osimertinib to placebo in patients with stage IB to IIIA, EGFR-mutated, resected NSCLC.

**Methods:** Based on the results obtained from the ADAURA trial, a Markov model with three-state was employed to simulate patients who were administered either osimertinib or placebo until disease recurrence or completion of the study period (3 years). Quality-adjusted life-years (QALYs), lifetime costs, and incremental cost-effectiveness ratio (ICER) were calculated with a willingness-to-pay (WTP) threshold of $150,000 per QALY. Both univariate and probabilistic sensitivity analyses were carried out to explore the robustness of the model.

**Results:** Osimertinib produced additional 1.59 QALYs with additional costs of $492,710 compared to placebo, giving rise to ICERs of $309,962.66/QALY. The results of the univariate sensitivity analysis indicated that the utility of disease-free survival (DFS), cost of osimertinib, and discount rate had the greatest impact on the outcomes. Probabilistic sensitivity analysis showed that osimertinib exhibited a 0% chance of being considered cost-effective for patients using a WTP threshold $150,000/QALY.

**Conclusion:** In our model, osimertinib was unlikely to be cost-effective compared to placebo for stage IB to IIIA, EGFR-mutated, completely resected NSCLC patients from the perspective of a U.S. payer at a WTP threshold of $150,000 per QALY.

## Introduction

Lung cancer is the leading cause of mortality among all types of cancers globally. In the United States, there are an estimated annual incidence of 235,760 cases and 131,880 deaths associated with this condition (Siegel et al., 2022). About 85% of lung cancer cases are classified as non-small cell lung cancer (NSCLC) (de Groot et al., 2018). Only 25%–30% of newly diagnosed NSCLC patients have a disease, which could be considered resectable, while the majority are diagnosed at an advanced stage, either metastatic or locally advanced (Le Chevalier, 2010). However, exclusive reliance on surgical resection may not lead to complete cure in a substantial number of early-stage NSCLC patients, due to the escalating probability of disease relapse concurrent with disease progression. Furthermore, even after the complete excision of localized or locoregional disease via surgical intervention, 30%–55% of patients who undergo resection will eventually experience the development of metastatic disease (Uramoto and Tanaka, 2014).

For a considerable period of time, platinum-based adjuvant chemotherapy has been widely adopted as the standard treatment for individuals with resectable stage II–IIIA disease. However, the observed survival benefits have been relatively modest, resulting in an approximate 5% increase in overall survival (OS) (Pignon et al., 2008). Moreover, studies have elucidated that among individuals diagnosed with early or locally advanced NSCLC, the ones with an epidermal growth factor receptor (EGFR) mutated disease are more likely to relapse after post-operative chemotherapy or other definitive interventions, compared with the EGFR wildtype counterpart (Takahashi et al., 2022).

Osimertinib is an oral third-generation EGFR tyrosine kinase inhibitor (EGFR-TKI), which has potent and selective inhibitory effects against both EGFR-TKI sensitizing mutations and EGFR p.Thr790Met resistance mutations (Cross et al., 2014). In the 2020 ADAURA primary analysis, adjuvant osimertinib demonstrated a substantial improvement in disease-free survival (DFS) compared to placebo for individuals with EGFR-mutated NSCLC who had undergone complete tumor resection, regardless of prior adjuvant chemotherapy. Importantly, no significant adverse events were observed (Wu et al., 2020). These findings from the ADAURA trial represent a major breakthrough in perioperative treatment for NSCLC, marking the first significant advancement in over a decade. As a result, adjuvant osimertinib has now received FDA approval for individuals with EGFR-mutated resected NSCLC.

After the release of the OS data from the ADAURA randomized clinical trial recently, noteworthy enhancements in OS were also discerned (Tsuboi et al., 2023). The 5-year OS rate in the osimertinib group was found to be 88%, in comparison to 78% in the placebo group (hazard ratio, 0.49; 95%CI, 0.34–0.70; *p* < 0.001). However, further investigation is necessary to assess the cost-effectiveness characteristics of osimertinib due to its relatively high price. Moreover, evaluating the cost-effectiveness of medical interventions could aid decision-makers and healthcare professionals in optimizing the allocation of limited healthcare resources.

Our study from the perspective of U.S. payers, aimed to assess the cost-effectiveness of osimertinib vs. placebo among the stage IB to IIIA, EGFR-mutated, completely resected NSCLC patients.

## Methods

### Participants and interventions

The fundamental clinical information was collected from the ADAURA trial, which was a globally conducted phase 3 trial that followed a double-blind, placebo-controlled design (Tsuboi et al., 2023). The study cohort included individuals who underwent surgical excision of primary tumors at stage IB, II, or IIIA NSCLC, bearing EGFR mutation characterized by either exon 19 deletion (Ex19del) or exon 21 codon p.Leu858Arg (L858R) point mutation. Patients were subjected to a random assignment in a 1:1 ratio, where they were either allocated to receive either oral osimertinib or a placebo for a duration of 3 years, or until the occurrence of disease recurrence or meeting a predefined criterion for discontinuation.

### Model construction

The TreeAge Pro 2022 software (TreeAge, Massachusetts, United States) was used to construct Markov model in order to assess the economic implications and clinical outcomes associated with osimertinib. Subsequently, statistical analysis was conducted utilizing R software (version 4.2.1). The model framework encompasses three distinct health states that are mutually exclusive: DFS, disease recurrence, and death (Supplementary Figure S1). In accordance with the findings of the ADAURA study, our model incorporates patients with a median age of 63 who underwent surgical excision of primary tumors. Following this procedure, two treatment alternatives are available for consideration: oral administration of either 80 mg osimertinib once daily or placebo. Osimertinib was administered for a maximum duration of 3 years or until disease recurrence in the study population.

Subsequent administration of anticancer therapeutics occurred in 67.1% of individuals who received osimertinib and 66.3% of individuals who received placebo within their respective study cohorts following disease recurrence (Supplementary Table S1). The subsequent chemotherapy regimen after disease recurrence was based on the PARAMOUNT trials, consisting of maintenance therapy with pemetrexed following induction therapy with pemetrexed plus cisplatin for four cycles (Paz-Ares et al., 2012).

In order to align with the chemotherapy cycle, we defined one cycle length in our model as a duration of 3 weeks. The time limit of 275 cycles was set based on the average life expectancy at birth of 78.8 years in the U.S. (Arias and Xu, 2022). The primary outcomes of our study encompassed overall costs, quality-adjusted life-years (QALYs), and incremental cost-effectiveness ratios (ICERs). Half-cycle correction and 3% annual discount rate were used in the calculation of cost and life expectancy (Lin et al., 2020) (Table 1).

**TABLE 1:** Model parameters and distributions.

Variables	Baseline values (references)	Range		Distribution
		Minimum	Maximum	
Log-logistic DFS survival model with osimertinib group	shape = 1.63351; scale = 112.73457	—	—	—
Gen-gamma DFS survival model with placebo group	mu = 2.88767	—	—	—
	sigma = 1.45261			
	Q = −1.30760			
Gen-gamma OS survival model with osimertinib group	mu = 4.36038	—	—	—
	sigma = 1.26750			
	Q = −3.13596			
Log-normal OS survival model with placebo group	meanlog = 4.83614	—	—	—
	sdlog = 1.01581			
Utility				
Disease-free survival	0.83 (Stewart EL et al., 2015)	0.67	0.99	Beta
Disease recurrence	0.74 (Stewart EL et al., 2015)	0.59	0.89	Beta
Drug cost ($^a^)				
Osimertinib/80 mg	566.64 (Drugs.com, 2022)	453.31	679.97	Gamma
Pemetrexed/10 mg	7.51 (CMS, 2023)	6.01	9.01	Gamma
Cisplatin/10 mg	3.17 (CMS, 2023)	2.54	3.80	Gamma
Administration cost per cycle ($^a^)	155.09 (CMS, 2023)	124.07	186.11	Gamma
Tumor imaging cost per cycle ($^a^)	249.48 (Ding et al., 2021)	199.58	299.38	Gamma
Laboratory testing cost per cycle ($^a^)	340.20 (Ding et al., 2021)	272.16	408.24	Gamma
The one-time cost of end-of-life care during the terminal stage ($^a^)	10187.64 (Liu et al., 2021)	8150.11	12225.17	Gamma
Physician visit cost per cycle ($^a^)	160.20 (CDCP, 2023)	128.16	192.24	Gamma
Best supportive care cost per cycle ($^a^)	481.57 (Liu et al., 2021)	385.26	577.88	Gamma
Patients’ body surface area, m^2^	1.82 (Lin et al., 2020)	1.46	2.18	Normal
Discount rate (%)	3 (Lin et al., 2020)	0	5	Fixed in PSA

DFS, disease-free survival; OS, overall survival; PSA, probabilistic sensitivity analyses.

^a^ US, dollar.

### Costs estimates

The evaluation of costs was carried out from the perspective of American third-party public healthcare payers. We considered health resource utilization and direct medical expenses, encompassing drug procurement, disease management, drug administration, and treatment-related adverse events (Table 1). The drug dosage was determined based on an average body surface area of 1.82 m^2^ (Goulart and Ramsey, 2011).

We extracted drug prices from the Centers for Medicare and Medicaid Services and Drugs.com (Drugs com, 2022). The expenses associated with the administration of medication, best supportive care, end-of-life palliative care, and disease management (which includes costs related to hospitalization, computed tomography, and laboratory examinations) were obtained from pre-existing databases that have been published previously (Lin et al., 2020; Ding et al., 2021; Liu et al., 2021; CDCP, 2023; CMS, 2023). Based on the ADAURA study, costs associated with computed tomography scans, laboratory tests, and physician visits were documented for both the osimertinib and placebo groups at weeks 12 and 24. These assessments were then conducted every 24 weeks over a period of 5 years, followed by annual evaluations. After the disease recurrence, the costs associated with administration, laboratory testing, and physician visits were documented during each treatment cycle for both chemotherapy and best supportive care. Additionally, the cost of computed tomography was recorded every two treatment cycles. To account for inflation and reflect the values of U.S.D. 2023, we employed the American Consumer Price Index (CPI) for cost adjustments. Specifically, we employed the Tom’s Inflation Calculator to inflate the costs to align with the year 2023 (Medical-care-inflation, 2022). We employed a willingness to pay (WTP) threshold of $150,000/QALY to analyze the outcomes (Neumann et al., 2014; Peng et al., 2021; Hu et al., 2022).

Analogous to traditional research methodologies, our primary focus is on severe treatment-related AEs (grade 3 or higher) that occur at an incidence rate of 5% or above. Milder AEs, on the other hand, typically do not require medical attention or result in significant expenses (Nafees et al., 2008; Su et al., 2021; Liu et al., 2022). In the ADAURA study, no severe treatment-related AEs occurred at a rate surpassing 5% (Tsuboi et al., 2023).

### Survival and progression transition estimates

The transition probability based on the DFS and OS curves of the ADAURA study was extrapolated utilizing the GetData Graph Digitizer software (version 2.22). The algorithm developed by Hoyle et al. was utilized to generate the simulated patient data (Hoyle and Henley, 2011). The data from curves were fitted to various survival functions such as exponential, log-logistic, log-normal, gengamma, gamma, Weibull, Gompertz, and distributions using the Akaike and Bayesian information criterion, aiming to achieve optimum fit (Supplementary Figure S2 and Supplementary Table S2). Each age group of the background death rates were assessed using U.S. life tables (Supplementary Table S3) (Arias E, 2020).

### Health-state utilities

The health utility for DFS, disease recurrence, and death were sourced from previous published investigations that were determine to be 0.83, 0.74, and 0, respectively (Stewart EL et al., 2015). Similar to conventional research approaches, the primary emphasis is placed on severe adverse events (grade ≥3) that manifest at an incidence rate of 5% or above (Kuznik et al., 2022). This is mainly because mild adverse reactions usually do not necessitate treatment or result in substantial treatment expenses. In the ADAURA study, no adverse events meeting the criteria of grade ≥3 and an incidence rate exceeding 5% were observed.

### Univariate and probabilistic sensitivity analyses

To explore the model’s robustness, we carried out probabilistic sensitivity analyses and oneway sensitivity analyses (Wang et al., 2021). We systematically adjusted clinical parameters within a range that accounted for plausible deviations of 20% from their baseline values in the univariate sensitivity analysis. These corresponding variations are visually presented in the tornado diagram. We employed 1,000 Monte Carlo simulations to perform a sensitivity analysis on the probability. This involved simultaneously and randomly varying preset parameters according to specific distribution patterns. The costs follow gamma distributions, while the proportion, and utility follow beta distributions (Table 1).

## Results

### Base case results

In the context of our Markov model, the estimated cumulative costs per patient over the lifetime horizon amounted to $620,436 for the osimertinib group and $127,726 for the placebo group. The osimertinib treatment resulted in 8.05 QALYs while the placebo treatment yielded 6.46 QALYs. As a result, individuals receiving osimertinib gained an increase of 1.59 QALYs but incurred an additional cost of $492,710 compared to the placebo group. This led to an ICER of $309,962.66/QALY, surpassing the predetermined WTP threshold of $150,000/QALY (Table 2).

**TABLE 2:** Base-case results of the model.

Group	Costs ($^a^)	△Costs ($^a^)	QALYs	△QALYs	ICER ($^a^/QALY)
Placebo	127,726	—	6.46	—	—
Osimertinib	620,436	492,710	8.05	1.59	309,962.66

ICER, incremental cost-effectiveness ratio; QALYs, quality-adjusted life-years.

^a^ US dollar.

### Sensitivity analysis

**FIGURE 1:**
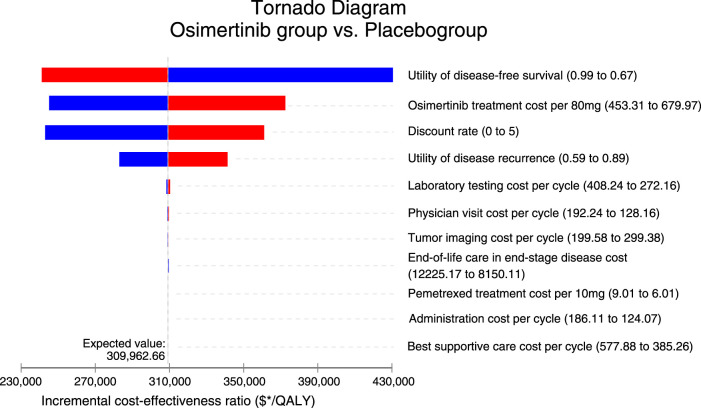
Tornado diagram for univariate sensitivity analyses. QALY, quality-adjusted life-year.

As illustrated in Figure 1, the tornado diagram reveals the prominent influence of specific parameters on the ICER, such as the utility of DFS, cost of osimertinib, and discount rate, utility of disease recurrence. Other variables have a minimal impact on the outcome. The absence of convergence between the generated ICER and WTP values, with all parameters varying within their respective ranges, serves as confirmation that the model outcomes maintain robustness. When the price of osimertinib drops to $282.86/80 mg, the ICER decreases to $150,000, matching the predetermined WTP threshold.

**FIGURE 2:**
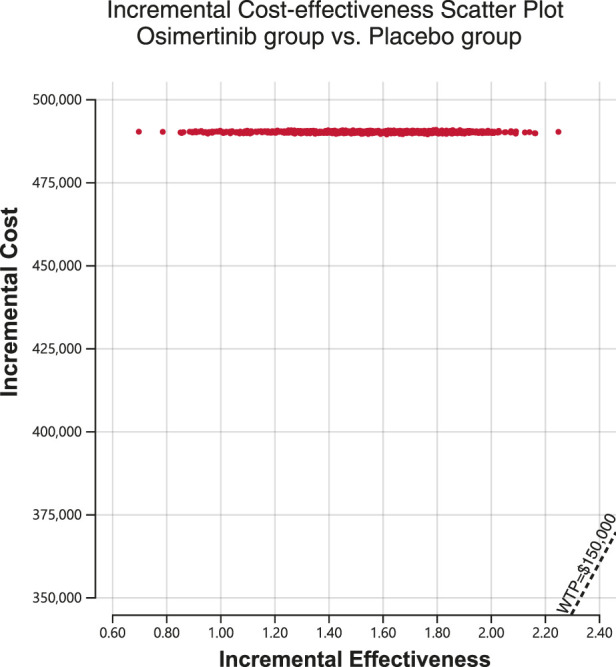
Incremental cost-effectiveness scatter plot diagram for osimertinib versus placebo. WTP, willingness-to-pay.

**FIGURE 3:**
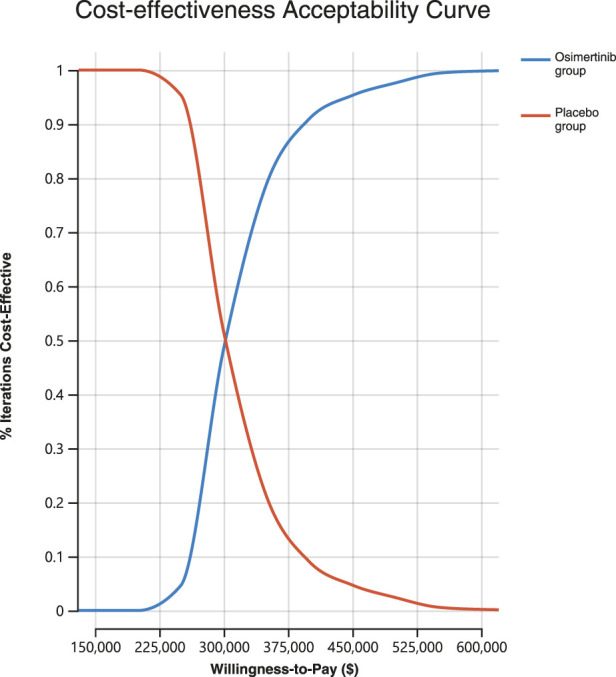
The cost-effectiveness acceptability curve for probabilistic sensitivity analyses.

A Monte Carlo simulation was conducted on a sample size of 1,000 individuals in order to investigate the spatial distribution of data points. The findings showed that all scatter points were situated in the first quadrant of the coordinate axis, suggesting that the use of osimertinib may result in a greater cost, albeit a higher number of QALYs gained. Furthermore, examination of Figure 2 demonstrated that all scatter points fell above the WTP line. Probabilistic sensitivity analysis showed that osimertinib exhibited a 0% chance of being considered cost-effective for patients using a WTP threshold $150,000 per QALY (Figure 3).

## Discussion

According to our model findings, the results of our base case analysis suggest that osimertinib exhibits superior health outcomes but yields higher costs in comparison to placebo among individuals with stage IB to IIIA, EGFR-mutated, completely resected NSCLC. The PSA results indicate that osimertinib is unlikely to be considered a cost-effective alternative, as it surpasses the predefined WTP threshold of $150,000 per QALY when compared to placebo.

Prior models have evaluated the cost-effectiveness of adjuvant osimertinib for individuals with EGFR-mutant resected NSCLC compared with placebo (Lemmon et al., 2022; Zhou et al., 2022; Verhoek et al., 2023). However, the cost-effectiveness analysis was based on the OS data extracted from AURA3 and FLAURA clinical trials, which were conducted in advanced NSCLC. The selection of these advanced NSCLC patients for the purpose of cost-effectiveness analysis was driven by the immaturity of OS data from the ADAURA trial at the time, imposing an inevitable bias, due to the discernible disparity in survival rates between patients with advanced disease and those classified as stage IB to IIIA. With the disclosure of OS data from the ADAURA trial, our study meticulously evaluated the cost-effectiveness attributes of adjuvant osimertinib by utilizing the most up-to-date information.

The influential factors in our model encompassed the utility value of DFS and disease recurrence. The adopted utility value in our analysis referred to the published data on health utility values of NSCLC patients with EGFR mutations. Specifically, patients with EGFR mutations who positively responded to osimertinib were assigned a utility value of 0.83, while those experiencing disease recurrence during treatment with TKIs (osimertinib, gefitinib, erlotinib) were assigned a utility value of 0.74 (Stewart EL et al., 2015; Paracha et al., 2018). To explore the impact of health utility value on our model, we conducted a sensitivity analysis by defining variable ranges for each utility value. Specifically, the range for DFS utility was set between 0.67 and 0.99, while the range for disease recurrence utility was set between 0.59 and 0.89. The results revealed that neither the highest nor lowest utility values made osimertinib cost-effective.

The costs of osimertinib were found to have a significant impact in our model’s sensitivity analyses. Despite variations in the sensitive variable within ±20% of the base price range of $453.31 to $679.97 per 80 mg, the ICERs remained above $150,000 per QALY, indicating a lack of cost−effectiveness. Meanwhile, the use of adjuvant osimertinib, with its proven efficacy, prolongs the duration of the expensive treatment for patients, thereby making the significant cost a crucial factor to consider. When the price of osimertinib drops to $282.86/80 mg, the ICER decreases to $150,000, matching the predetermined WTP threshold. Therefore, the most practical approach to achieve cost-effectiveness in adjuvant treatment is to reduce the prices of osimertinib. Despite the approval of adjuvant osimertinib as a new step towards providing a more effective adjuvant therapy strategy for resected, EGFR-mutated NSCLC, it is important to mention that, from the perspective of third-party public healthcare payers, concerns about affordability and sustainability due to the high pricing of antitumor agents. Furthermore, from a patient perspective, the high cost may expose patients to a significant risk of economic toxicity as they may have to bear the financial burden of self-paying medical costs that may not be fully covered by health insurance. Evidence has shown that economic toxicity leads to economically disadvantaged patients discontinuing, postponing, or abandoning their therapeutic regimens (Carrera et al., 2018). It is equally important for healthcare systems to guarantee equal access to innovative treatments in order to reduce financial harm (de Souza and Conti, 2017). For instance, taking a strategic approach to improve cost-effectiveness could involve negotiating the pricing and coverage of osimertinib, resulting in an effective and prudent intervention.

There were certain limitations in this study. Firstly, it is important to extend the survival curve in order to obtain comprehensive survival outcomes within our framework. However, the reconstructed survival curves did not fully match the actual ones. Nonetheless, the aim of adjusting the transition probability is to closely approximate the real results. Secondly, in accordance with the majority of previous studies, we exclusively focus on AEs of grade ≥3 and with an occurrence rate of ≥5%. Consequently, no associated costs related to AEs were documented, potentially leading to an underestimation of the ICER. It is noteworthy that all AEs observed in the ADAURA trial had an incidence rate ≤2% and were reversible upon temporary suspension of treatment, thereby exerting minimal impact on the study outcomes. Thirdly, treatment decisions were limited in the disease recurrence state due to variations in clinical practice. We did not include local lesion radiotherapy, surgeries or other treatment methods, which may limits the real world applicability once individuals enter this state. Despite these limitations, our study provides valuable insights into the cost-effectiveness of adjuvant osimertinib in the treatment of EGFR-mutated NSCLC. The findings highlight the need for careful consideration of both clinical outcomes and costs when making treatment decisions. Future research should focus on addressing the limitations mentioned above and further evaluating the long-term cost-effectiveness of adjuvant osimertinib.

## Conclusion

From the perspective of a U.S. payer, osimertinib was unlikely to be cost-effective compared to placebo for patients with stage IB to IIIA, EGFR-mutated, completely resected NSCLC at a WTP threshold of $150,000/QALY. Our analysis suggests that while osimertinib exhibits superior health outcomes compared to placebo, however, it is not cost-effective at its current price. Efforts should be made to negotiate the pricing and coverage of osimertinib to improve its cost-effectiveness and ensure equal access to innovative treatments for all patients.

## Data Availability

The original contributions presented in the study are included in the article/Supplementary Material, further inquiries can be directed to the corresponding authors.
