# Supplementary material for: Osimertinib in the treatment of resected EGFR-mutated non-small cell lung cancer: a cost-effectiveness analysis in the United States
Source: Front Pharmacol. 2024 Mar 28;15:1300183. doi: 10.3389/fphar.2024.1300183 (PMC11007098; doi:10.3389/fphar.2024.1300183)
Supplement: Supplementary file 1 [file Table1.DOCX]

**Table S1** **Subsequent Treatment Regimen Selection for Patients Following Disease Relapse**

| **Treatment regimen** | **Osimertinib group** | **Placebo group** |
| --- | --- | --- |
| Osimertinib | 0.408 | 0.429 |
| Chemotherapy | 0.263 | 0.234 |
| Best Supportive Care | 0.105 | 0.223 |
| Ceased treatment | 0.224 | 0.114 |

**Table S2 AIC and BIC statistics for alternate parametric survival distributions**

| **Distribution** | **DFS** | | | | **OS** | | | |
| --- | --- | --- | --- | --- | --- | --- | --- | --- |
|  | **Osimertinib group** | | **Placebo group** | | **Osimertinib group** | | **Placebo group** | |
|  | **AIC** | **BIC** | **AIC** | **BIC** | **AIC** | **BIC** | **AIC** | **BIC** |
| Exponential | 1123.666 | 1127.492 | 2064.816 | 2068.654 | 614.9988 | 618.8248 | 1070.410 | 1074.248 |
| Gamma | 1110.637 | 1118.289 | 2065.354 | 2073.030 | 588.3622 | 596.0142 | 1046.364 | 1054.039 |
| Gengamma | 1112.442 | 1123.920 | 2005.839 | 2017.352 | 585.1407 | 596.6187 | 1047.106 | 1058.619 |
| Gompertz | 1117.070 | 1124.722 | 2034.802 | 2042.477 | 597.0133 | 604.6653 | 1055.248 | 1062.924 |
| Weibull | 1111.297 | 1118.949 | 2062.359 | 2070.034 | 589.8247 | 597.4767 | 1047.514 | 1055.190 |
| Log-logistic | 1110.092 | 1117.744 | 2037.432 | 2045.107 | 589.1716 | 596.8236 | 1046.509 | 1054.184 |
| Log-normal | 1113.774 | 1121.426 | 2024.572 | 2032.248 | 586.3393 | 593.9913 | 1045.116 | 1052.792 |

AIC, Akaike information criterion; BIC, Bayesian information criterion; DFS, disease-free survival; OS, overall survival.

**Table S3 Background mortality rate**

| **Age** | **Background mortality rate** | **Age** | **Background mortality rate** | **Age** | **Background mortality rate** |
| --- | --- | --- | --- | --- | --- |
| 26 | 0.000968 | 51 | 0.004484 | 76 | 0.03287 |
| 27 | 0.000994 | 52 | 0.004874 | 77 | 0.036315 |
| 28 | 0.001024 | 53 | 0.005302 | 78 | 0.040253 |
| 29 | 0.001058 | 54 | 0.005771 | 79 | 0.044908 |
| 30 | 0.001095 | 55 | 0.006274 | 80 | 0.049974 |
| 31 | 0.001132 | 56 | 0.006793 | 81 | 0.055475 |
| 32 | 0.001171 | 57 | 0.007321 | 82 | 0.061509 |
| 33 | 0.001213 | 58 | 0.007854 | 83 | 0.068675 |
| 34 | 0.00126 | 59 | 0.008403 | 84 | 0.076701 |
| 35 | 0.001319 | 60 | 0.008999 | 85 | 0.085469 |
| 36 | 0.001389 | 61 | 0.009652 | 86 | 0.095935 |
| 37 | 0.001467 | 62 | 0.010341 | 87 | 0.107533 |
| 38 | 0.00155 | 63 | 0.011056 | 88 | 0.120347 |
| 39 | 0.001639 | 64 | 0.011804 | 89 | 0.134457 |
| 40 | 0.001743 | 65 | 0.012598 | 90 | 0.149939 |
| 41 | 0.001864 | 66 | 0.013484 | 91 | 0.166861 |
| 42 | 0.002001 | 67 | 0.014501 | 92 | 0.185276 |
| 43 | 0.002159 | 68 | 0.015701 | 93 | 0.205223 |
| 44 | 0.002345 | 69 | 0.017146 | 94 | 0.226719 |
| 45 | 0.002547 | 70 | 0.018855 | 95 | 0.24976 |
| 46 | 0.002778 | 71 | 0.020762 | 96 | 0.274312 |
| 47 | 0.003059 | 72 | 0.022816 | 97 | 0.300311 |
| 48 | 0.003391 | 73 | 0.02501 | 98 | 0.327661 |
| 49 | 0.003753 | 74 | 0.027353 | 99 | 0.356235 |
| 50 | 0.004118 | 75 | 0.029897 | 100+ | 1 |
