# Supplementary material for: Osimertinib in the treatment of resected EGFR-mutated non-small cell lung cancer: a cost-effectiveness analysis in the United States
Source: Front Pharmacol. 2024 Mar 28;15:1300183. doi: 10.3389/fphar.2024.1300183 (PMC11007098; doi:10.3389/fphar.2024.1300183)
Supplement: Supplementary file 2 [file DataSheet1.pdf]

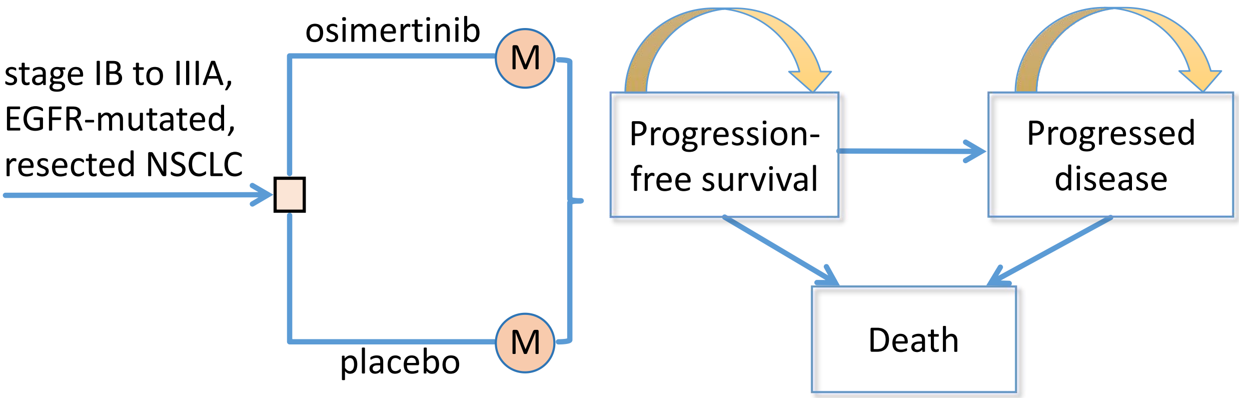

Figure S1 Markov state transition diagram

A

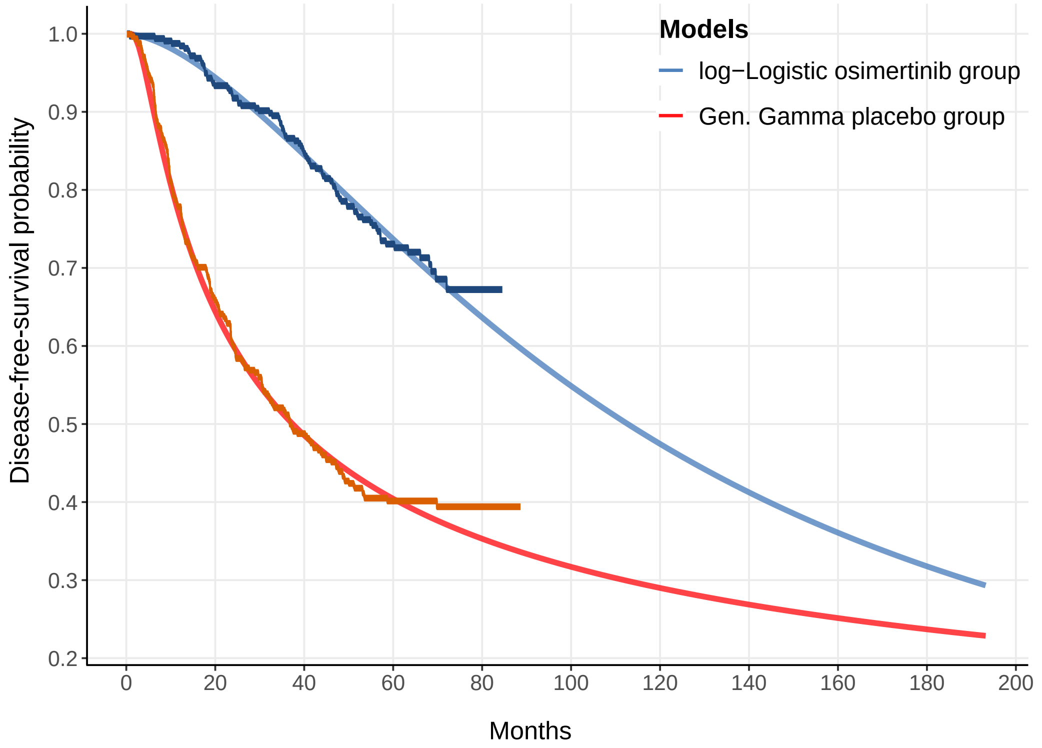

B

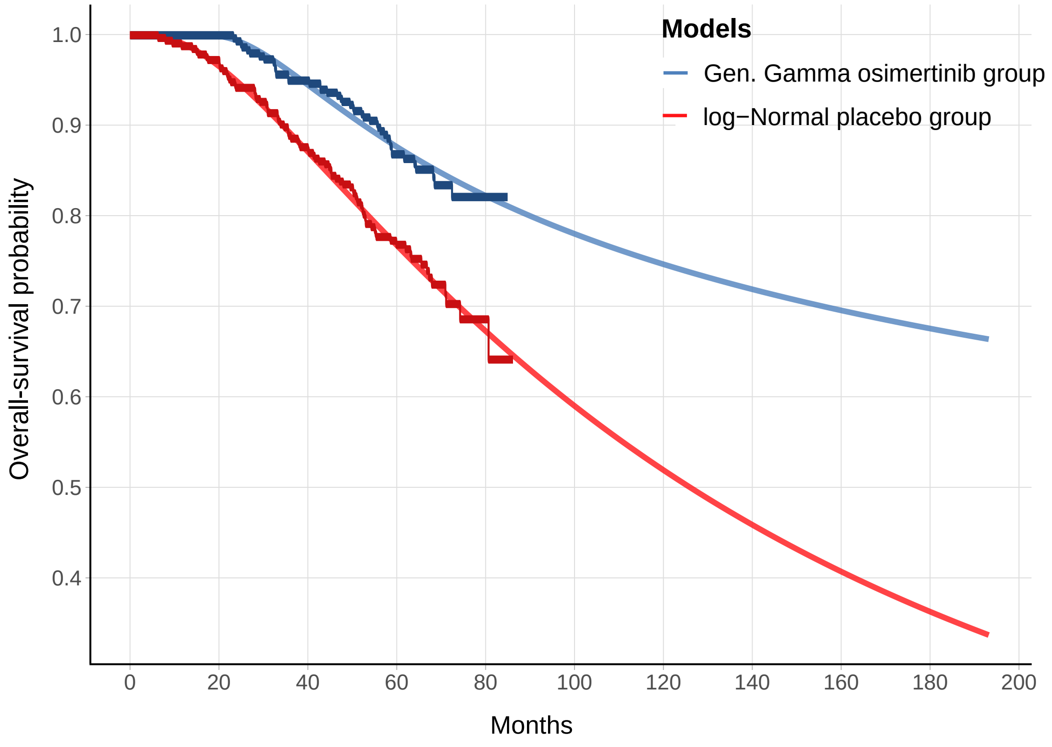

Figure S2 Disease-free-survival curves for the original trial and model estimated data (A) and overall-survival curves for the originaltrial and estimated data (B)
